# Rheological properties and damage-control mechanism of oil-based drilling fluid with different types of weighting agents

**DOI:** 10.1098/rsos.180358

**Published:** 2018-07-25

**Authors:** Peng Xu, Mingbiao Xu, Zhengwu Tao, Zhihong Wang, Ting Huang

**Affiliations:** 1 College of Petroleum Engineering, Yangtze University, Wuhan, China; 2 Hubei Cooperative Innovation Center of Unconventional Oil and Gas, Yangtze University, Wuhan, China; 3 Research Institute of Exploration and Development, Tarim Oilfield Company, PetroChina, Korla, China; 4 Petroleum Engineering & Technology Research Institute, North China oil and gas branch, Sinopec, Zhengzhou, China

**Keywords:** oil-based drilling fluid, rheology, weighting agent, formation damage, micromorphology

## Abstract

The great amount of solid particles contained in a weighting agent is a major cause of the problems in both rheology properties and damage control mechanism of an oil-based drilling fluid (OBM). Therefore, a proper type of weighting agent can be a solution for the application of OBM. In this study, three weighting agents that have been commonly used with OBM, namely, standard barite, submicron barite and superfine manganese ore, are studied. Rheological properties of OBM and the degree of formation damage are assessed with regard to the three weighting agents. The agents are also studied in aspects of particle size, micromorphology, filtration loss and wall-building property, acid dissolution efficiency of mud cake, lubricity and sedimentation stability to analyse the effects of the agents on rheological properties and the degree of damage as well as to figure out the mechanism of rheology control and damage control. For the OBM, there is a mutual effect between rheological stability and the degree of damage. In consideration of the agents' properties, we can enhance the rheological stability of the OBM and control the degree of formation damage by properly selecting particle size, using acid-soluble materials and forming the mud cake with ultra-low permeability that can easily be cleared away.

## 1. Introduction

An oil-based drilling fluid has wide application in an unstable formation, deep well and ultra-deep well. It is an efficient tool to reduce drilling accidents and raise the drilling speed [1]. In most applications, a number of weighting agents are used to increase the density of the oil-based drilling fluid [2]. However, the high density can cause a series of problems such as high solid content, high viscosity and high friction coefficient [3,4]. Nowadays, keeping good rheology of the oil-based drilling fluid and reducing the reservoir damage have been increasingly concerned in drilling projects [5–7].

Standard barite and a small amount of superfine barite have been the major weighting agents for the oil-based drilling fluid over the years. In an application where the density is high, however, the suspension of the standard barite is not stable and can easily be affected by temperature and pressure, which will seriously affect the drilling speed and also increase the probability of accidents [8]. By far, many studies on barite surface modification have been conducted to improve the weighting ability of the barite, such as increasing the zeta potential on the barite's surface, enhancing its lipophilicity/hydrophilicity and strengthening its suspension stability [9,10]. For the drilling fluid with high density, however, a large amount of barite will cause the problem of rheological stability. In recent years, many weighting agents including the powders of limestone, iron ore, ilmenite, manganese ore and galena have been applied [11], but they have some defects: limestone is applicable for low density; the magnetism of iron ore has great effect on underground measurement; manganese ore needs to be used together with barite; and galena is costly though it is applicable for super high density [12–14].

The usage of a great amount of weighting agent will worsen the rheology, filtration loss and wall-building property, and lubricity of the oil-based drilling fluid [15]. The rheological stability of the oil-based drilling fluid with high density used with different types of weighting agent still needs to be studied. The rheology of the oil-based drilling fluid with high density is in fact an issue of the stability of a water-in-oil emulsion, which is an extremely complex disperse system and its stability is affected by liquid-phase properties, particulate composition, membrane structure and other factors [16,17]. The most important factor that affects the stability of water-in-oil emulsion is the stability at the oil–water interface, and the oil–water interfacial film is mainly composed of emulsifying agent, asphaltene, gelatin and solid-phase particles, among which the solid-phase particles are evenly dispersing in the oil and spreading to the interface, at which there is an equilibrium between lipophilicity and hydrophilicity [18,19]. The weighting agent is the major source of solid-phase particles. The thermodynamic instability of the oil-based drilling fluid relates to different types and amounts of weighting agent, which results in rheological instabilities of the drilling fluid such as layering, flocculation, coalescence and phase separation [20,21]. A wetting agent is usually added into the oil-based drilling fluid to modify the surface wettability of the barite and make it lipophilic, so as to make the emulsion more stable. Oil-based drilling fluids that have stable rheological properties have also been widely studied to reduce the negative effects of weighting agent, temperature, pressure and other factors [22].

The oil-based drilling fluid contains a great number of solid-phase particles due to the usage of a weighting agent, and the plugging of these particles is a core factor for the formation damage [23]. In comparison with water-based drilling fluid, the damage caused by the solid plugging in oil-based drilling fluid is mainly due to the hydrophilic wettability of the weighting agent particles. In an unstable rheological state, the hydrophilic particles will agglomerate into liquid drops with bigger sizes under the effect of the continual water-phase aggregation, which will change both the dispersity of the weighting agent and the size of the liquid drops, resulting in a coexistence of solid plugging and emulsion plugging [24,25]. Consequently, the permeability is not easy to recover after the drilling is finished. In studying damage control of a drilling fluid with high density, the key is the control of mud cake quality and the recovery rate of permeability [6]. To control mud cake quality is to make sure that the solid-phase particles can form into a dense mud cake, and to keep a high recovery rate of permeability is to make sure that the particles in the oil-based drilling fluid can efficiently flow back [3]. The usage of an acid-soluble weighing agent (e.g. iron ore and manganese ore) in association with pickling measures is effective to raise the recovery rate of permeability, and the super small particle size can, to some extent, improve the rheological stability of the oil-based drilling fluid and also reduce the formation damage [26].

The damage control of the oil-based drilling fluid depends partly on its rheological stability. In this article, the rheology of the oil-based drilling fluid and the damage of the steel core are assessed at different amounts of three weighting agents that have been commonly used in oil-based drilling fluid, namely, standard barite, submicron barite and superfine manganese ore. The effects of the weighting agents' particle size and micromorphology have also been analysed. With respect to filtration loss and wall-building property, acid dissolution efficiency of the mud cake, lubricity and sedimentation stability of the oil-based drilling fluid, we have revealed the mechanism of the weighting agents to control the rheological properties and the formation damage, with the intention of providing some useful information for the use of weighting agents in oil-based drilling fluid.

## 2. Experimental materials and methods

### 2.1. Materials and apparatus

*Experimental materials*: Standard barite (4.3 g cm^−3^, Guizhou, China); millimetre–micron barite powder (4.3 g cm^−3^, Guizhou, China); superfine manganese ore powder (4.6 g cm^−3^, Tarim Oilfield of China National Petroleum Corporation (CNPC)); agents for treating oil-based drilling fluid, including diesel, organic soil, emulsifying agent, fluid-loss agent and rheology modifier (Jinzhou Jiahua Science and Technology Ltd); steel core (304 steel); the core of matrix strata (dense sandstone with permeability less than 0.1 × 10^−3^ µm^3^) (Keshen Block of Tarim Oilfield of CNPC).

*Experimental apparatus*: Laser particle size analyser; electrical stability tester (Qingdao Chuangmeng Apparatus); field emission scanning electron microscope SU-8010 (Hitachi); high-temperature and high-pressure rheometer (FEI); lubrication coefficient tester; centrifugal machine; multifunctional core dynamic damage tester.

*Formula of the oil-based drilling fluid*:

1#: base oil + 1.0% organic soil + 1.5% primary emulsifier + 2.1% secondary emulsifier + 20% CaCl_2_ brine (80/20) + 2.5% fluid-loss agent + 2.5% lime + 0.5% wetting agent + weighting agent. To ensure the correctness of the experimental results, when the experiment did not specify which drilling fluid is to be used, the oil-based drilling fluid is used as the 1# formulation.
2#: base oil + 1.0% organic soil + 3.2% primary emulsifier + 0.8% secondary emulsifier + 20% CaCl_2_ brine (80/20) + 1.0% fluid-loss agent + 1.5% lime + 1.0% wetting agent + weighting agent.
3#: base oil + 2.4% organic soil + 1.2% primary emulsifier + 1.2% secondary emulsifier + 20% CaCl_2_ brine (80/20) + 3.0% fluid-loss agent + 2.0% lime + 1.2% wetting agent + weighting agent.

### 2.2. Experimental method

*Rheological properties*: The prepared oil-based drilling fluid is weighted by using standard barite, submicron barite and superfine manganese ore, respectively. Different amounts of the same weighting agent, i.e. 200, 400 and 600 g, are added into the oil-based drilling fluid to test the rheological properties of the fluid. The compound of different weighting agents: different amounts of the compound of standard barite and superfine manganese ore that are mixed in the ratio of 1 : 1 are added into the oil-based drilling fluid to test the changes of the rheological properties.

*Damage assessment*: In view of the fact that acidification is a common measure for increasing production, we carry out the assessment in two ways based on the consideration whether the weighting agent is acid-soluble or not. The flow-back pressure and damage rate of both the steel core and the artificially fractured matrix core are tested after they have been damaged by the oil-based drilling fluid. The experiment procedure without acid dissolution is: kerosene displacement—inverse displacement of oil-based drilling fluid—kerosene displacement. The experiment procedure with acid dissolution is: kerosene displacement—inverse displacement of oil-based drilling fluid—inverse displacement of acid solution—kerosene displacement.

## 3. Results

### 3.1. Rheology

**Figure 1.**
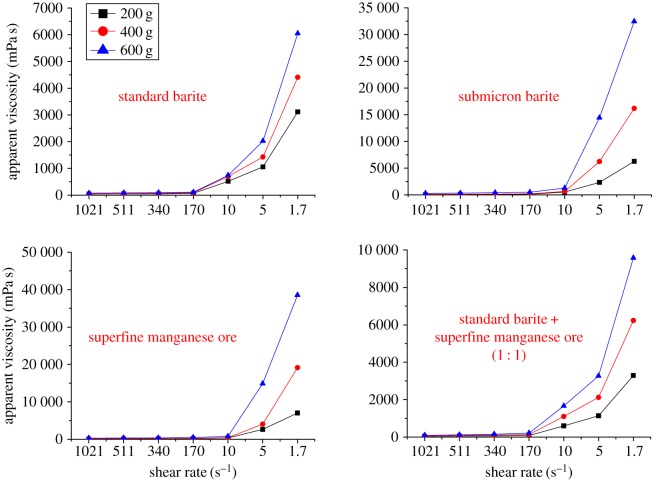
Relationship between shear rate and apparent viscosity of different weighting agents.

**Figure 2.**
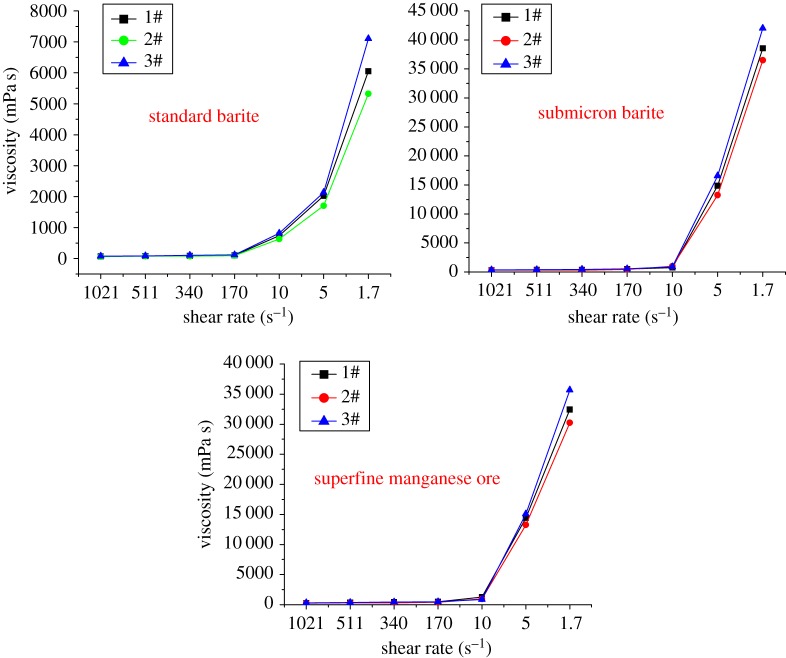
Rheological test results of different oil-based drilling fluids.

**Figure 3.**
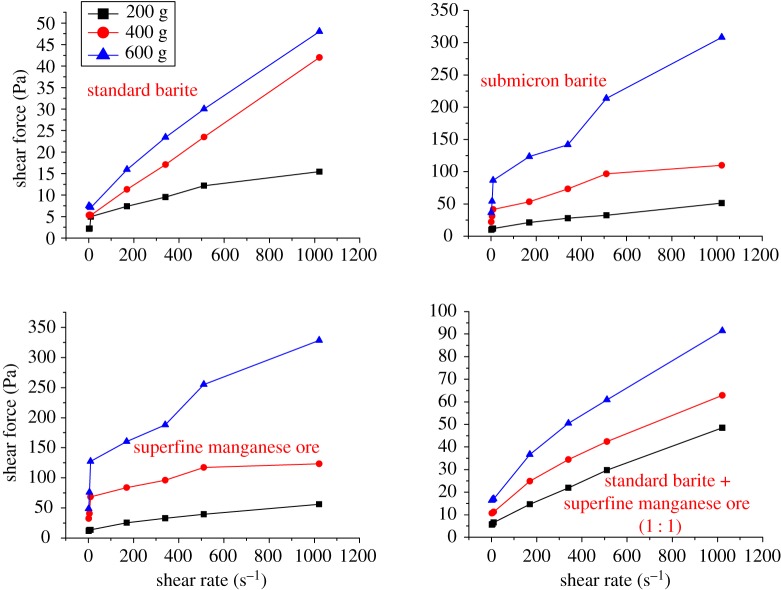
Relationship between shear rate and shear force of different weighting agents.

Rheology reflects the flow and the deformation of a drilling fluid under external forces. It is a basic performance index and also a key property ensuring that the drilling fluid can suspend and carry away the drilling cuttings, and clean the downhole and the borehole, and can drill safely and quickly. In accordance with the rheology-based assessment, we have obtained the relationship between shear rate and apparent viscosity (figure 1) and the relationship between shear rate and shear force (figure 3) of different weighting agents. To test the rheological properties of different oil-based drilling fluids, the evaluation of the rheological properties of three different oil-based drilling fluids was carried out after adding 600 g of different weighting agents; the results are shown in figure 2.

#### 3.1.1. Relationship between shear rate and apparent viscosity

Figure 1 shows the changing trends of apparent viscosity at different shear rates of 1# oil-based drilling fluid. Seen from the relationship between shear rate and apparent viscosity: the viscosity increases as the shear rate decreases, which is consistent with the basic change of the drilling fluid [7,19]. The apparent viscosity is ranked as: standard barite < compound < superfine manganese ore < submicron barite.

The experimental data of figure 2 show that the influence trend of the weighting agents on the rheological properties of different types of oil-based drilling fluids is consistent, the differences in rheological properties are within the allowable range and the differences between each other are caused by the difference between the treatment agents.

#### 3.1.2. Relationship between shear rate and shear force

Figure 3 shows the changing trends of shear force at different shear rates. Seen from the relationship between shear rate and shear force, the oil-based drilling fluid with the oil-to-water ratio of 80/20 shows a flow pattern of a plastic fluid when 200 g of weighting agent is added into the fluid. With increasing the weighting agent, the drilling fluid becomes more like a pseudoplastic fluid.

### 3.2. Damage degree

The degree of damage reflects the effect of the oil-based drilling fluid on the permeable capability of reservoir rocks, and permeability is the most direct reflection of the permeable capability. In consideration of the experimental impact of different cores, we use a steel core and matrix core taken from Keshen Block of Tarim Oilfield to carry out the experiments. Fractures are artificially created to the matrix core because its permeability is very low, and the permeability of the matrix core with artificial fractures is 5–10 × 10^−3^ µm^3^. Two experimental procedures (i.e. with and without acid dissolution) are performed. Table 1 presents the experimental results of the steel core and table 2 the experimental results of the matrix core with artificial fractures.

**Table 1.** Assessment of the recovery rate of permeability of steel cores.

experiments	weighting agent	core	pressure difference of flow-back (MPa)	recovery rate (%)	permeability before damage (10^−3^ µm^2^)	permeability after damage (10^−3^ µm^2^)
without acid dissolution	standard barite	1	0.01	90.56	1.2145	1.0999
		2	0.01	91.68	0.9678	0.8879
	submicron barite	1	0.01	93.45	0.8745	0.8172
		2	0.01	94.03	0.8642	0.8126
	superfine manganese ore	1	0.01	96.24	1.1156	1.0737
		2	0.01	95.38	0.6574	0.627
	standard barite + superfine manganese ore	1	0.01	92.14	0.7687	0.7083
		2	0.01	92.54	0.9382	0.8682
with acid dissolution	standard barite	1	0	93.65	0.7885	0.7384
		2	0	95.36	0.9853	0.9396
	submicron barite	1	0	97.12	0.7712	0.749
		2	0	95.14	0.9631	0.9163
	superfine manganese ore	1	0	99.93	0.8954	0.8948
		2	0	102.14	0.9375	0.9576
	standard barite + superfine manganese ore	1	0	95.36	0.8979	0.8562
		2	0	98.47	0.9937	0.9785

**Table 2.** Assessment of the recovery rate of permeability of matrix cores with artificial fracture.

experiments	weighting agent	core	pressure difference of flow-back (MPa)	recovery rate (%)	permeability before damage (10^−3^ µm^2^)	permeability after damage (10^−3^ µm^2^)
without acid dissolution	standard barite	RS1	2.27	36.98	0.7885	0.2916
		RS4	2.12	42.58	0.8413	0.3582
	submicron barite	RS5	1.79	47.85	0.6987	0.3343
		RS8	1.99	39.47	0.8524	0.3364
	superfine manganese ore	RS2	1.84	52.03	0.9638	0.5015
		RS7	2.02	37.18	0.7986	0.2969
	standard barite + superfine manganese ore	RS6	1.88	49.78	0.9741	0.4849
		RS9	2.28	45.23	0.9328	0.4219
with acid dissolution	standard barite	RS10	1.49	62.09	0.8657	0.5375
		RS11	1.53	53.98	0.8714	0.4704
	submicron barite	RR21	1.32	70.25	0.7967	0.5597
		RR24	1.16	74.36	1.0127	0.753
	superfine manganese ore	RR26	0.08	94.63	0.9634	0.9117
		RR27	0.12	98.14	0.8429	0.8272
	standard barite + superfine manganese ore	RR23	0.46	92.12	0.9183	0.8459
		RR25	0.67	94.65	0.9573	0.9061

As can be seen from the assessment of the damage to both the steel core and the natural core caused by the oil-based drilling fluid, the steel core recovers better than the natural core. The reason is that the fractures of the steel core are artificially created and the steel core is not as coarse as the natural core, which contributes to a higher recovery rate of permeability. In the experiment without acid dissolution, the degree of damage follows the distribution of the particle size. This is because the permeation area becomes decreased due to the damage, and the particle size distribution is greatly related to the amount and depth of the oil-based drilling fluid that permeates into the cores. In the experiment with acid dissolution, the effect of the oil-based drilling fluid weighted by superfine manganese ore on the permeability of the core is much lower than that of the drilling fluid weighted by acid-soluble barites. In general, acid dissolution can greatly help recover the permeability of the core and efficiently protect the reservoir of the oil-based drilling fluid.

To test the rheological properties of different oil-based drilling fluids, the evaluation of the core damage of three different oil-based drilling fluids was carried out after adding 600 g of different weighting agents; the results are shown in tables 3 and 4. The experimental data show that the influence of weighting agents on the reservoir protection ability of different oil-based drilling fluids is consistent.

**Table 3.** Recovery rate of permeability of artificial fractured core (2# oil-based drilling fluid).

experiments	weighting agent	core	pressure difference of flow-back (MPa)	recovery rate (%)	permeability before damage (10^−3^ µm^2^)	permeability after damage (10^−3^ µm^2^)
without acid dissolution	standard barite	ADD1	1.85	44.49	0.9635	0.4287
		ADD2	1.94	31.77	1.2521	0.3978
	submicron barite	ADD3	1.63	38.88	0.8654	0.3365
		ADD4	1.57	47.52	0.7873	0.3741
	superfine manganese ore	ADD5	1.54	45.59	0.9325	0.4251
		ADD6	1.51	52.94	0.9637	0.5102
with acid dissolution	standard barite	ADD7	1.49	56.18	0.9745	0.5475
		ADD8	1.58	57.83	0.8563	0.4952
	submicron barite	ADD9	1.36	68.41	0.9211	0.6301
		ADD10	1.21	61.61	1.1012	0.6784
	superfine manganese ore	ADD11	0.13	95.59	0.9632	0.9207
		ADD12	0.11	96.88	0.8741	0.8468

**Table 4.** Recovery rate of permeability of artificial fractured core (2# oil-based drilling fluid).

experiments	weighting agent	core	pressure difference of flow-back (MPa)	recovery rate (%)	permeability before damage (10^−3^ µm^2^)	permeability after damage (10^−3^ µm^2^)
without acid dissolution	standard barite	ADD11	2.52	35.51	1.1134	0.3954
		ADD12	2.46	33.59	1.2547	0.4214
	submicron barite	ADD13	2.11	38.11	0.8548	0.3258
		ADD14	2.23	36.79	0.9631	0.3543
	superfine manganese ore	ADD15	1.98	48.55	0.8267	0.4014
		ADD16	2.01	45.91	0.7968	0.3658
with acid dissolution	standard barite	ADD17	1.99	55.19	0.9257	0.5109
		ADD18	1.89	63.06	0.7459	0.4704
	submicron barite	ADD19	1.74	55.86	0.8569	0.4787
		ADD20	1.63	70.99	0.9646	0.6848
	superfine manganese ore	ADD21	0.22	92.99	1.1039	1.0265
		ADD22	0.27	91.48	0.8853	0.8099

#### 3.2.1. Steel core

Table 1 shows the permeability recovery rate of steel cores using different weighting agents. The results show that after the core damage experiments, the pressure difference of the steel core is very small, and acid solution and non-acid solution have little influence on the permeability recovery rate of the steel core, and the recovery rate of the permeability is above 90%.

#### 3.2.2. Matrix core with artificial fractures

Table 2 presents the permeability recovery rate of matrix cores using different weighting agents. The experimental results show that the recovery rate of core permeability with acid solution is much greater than that with non-acid solution.

## 4. Discussion

### 4.1. Analysis of particle size and micromorphology of weighting agent

#### 4.1.1. Analysis of particle size

**Figure 4.**
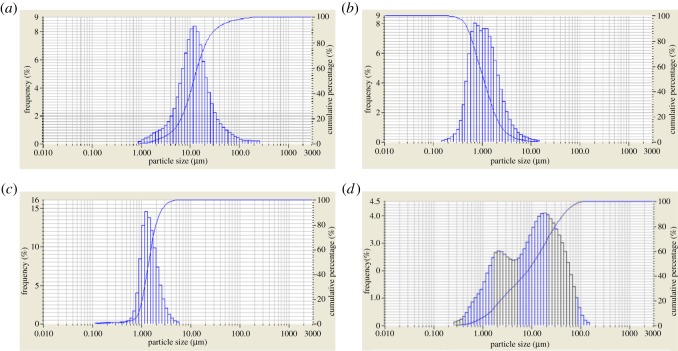
Particle size analysis of weighting agents. (*a*) Standard barite, (*b*) submicron barite, (*c*) superfine manganese ore and (*d*) standard barite + superfine manganese ore.

Analysis of particle size is an effective means to observe microscopic properties of a weighting agent. According to the analysis, we are able to distinguish the distributions of different particle sizes and, to some extent, predict potential damage to permeation channels of the reservoir caused by the weighting agent particles. To observe micromorphology, the three weighting agents are, respectively, added into 0.6% sodium hexametaphosphate solution to perform the ultrasonic dispersion for 5 min. The laser particle size analyser (LA-950A2) is used to measure the size distributions of the three weighting agents. For the purpose of accuracy, each sample is tested three times. Figure 4 shows the particle size distributions of standard barite, submicron barite, superfine manganese ore, and the compound of standard barite and superfine manganese ore (1 : 1).

As shown in figure 4, the size of the standard barite is much larger than the sizes of both the submicron barite and the superfine manganese ore. Most of the standard barite particles have a size above 10 µm, while the median sizes of the submicron barite and the superfine manganese ore fall in the range of 1.0–1.5 µm. The assessment of apparent viscosity suggests that the apparent viscosity is ranked as: standard barite < compound weighting agent < superfine manganese ore < submicron barite, which is the same as in the analysis of particle size. The reason for this ranking is that: for median size distribution and mean size distribution, the standard barite is the largest, and the compound system shows a multimodal distribution, while the mean size of superfine manganese ore is close to that of submicron barite, which is about one-tenth of the size of standard barite. Particles with a size of 1 µm are likely to form structural viscosity in oil-based drilling fluid, which, therefore, makes the viscosity greater.

The analysis of particle sizes of the three weighting agents shows that: the distribution intervals of submicron barite and superfine manganese ore particles are much narrower than those of the other two agents. If submicron barite or superfine manganese ore with this size is used, then structural viscosity will be formed in the oil phase. This result is consistent with the changing trend of the rheology, i.e. the oil-based drilling fluid becomes more stable. On the other hand, however, this is easy to result in flocculation if the system is not well controlled, which has great impact on the performance of the drilling fluid and also can cause damage to the reservoir. Standard barite is a proper weighting agent for drilling fluid. When acid dissolution is needed to remove the plug, then a small amount of superfine manganese ore can be added into the fluid.

#### 4.1.2. Micromorphology

**Figure 5.**
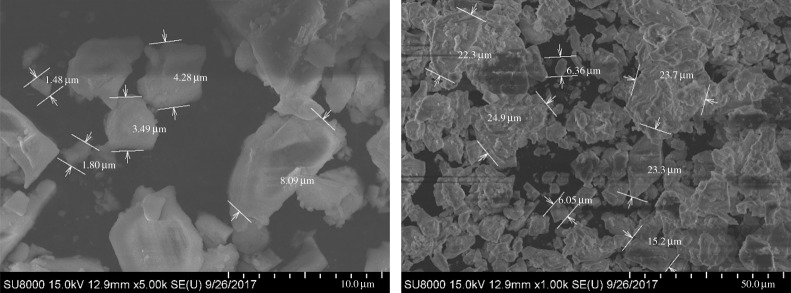
SEM images of standard barite.

**Figure 6.**
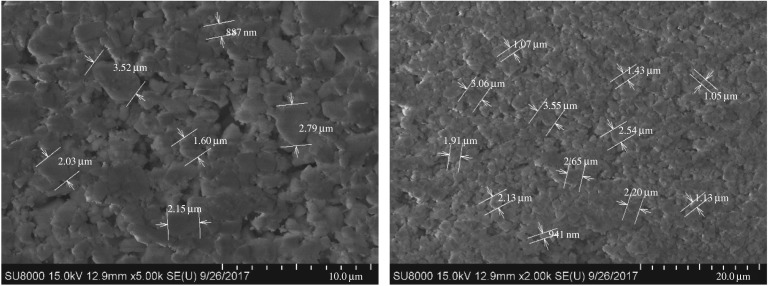
SEM images of submicron barite.

**Figure 7.**
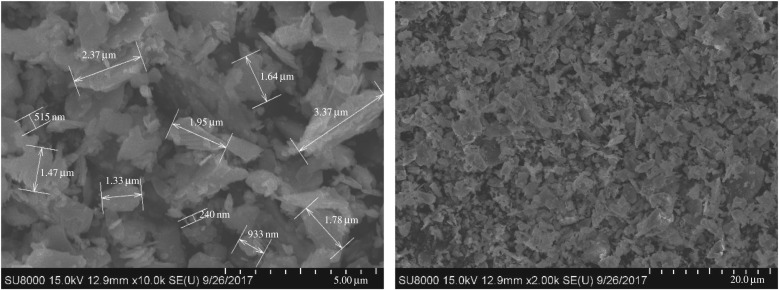
SEM images of superfine manganese ore.

The weighting agent has a great effect on the rheological properties of a drilling fluid as well as on formation damage. The size and morphology of the weighting agent decide its dispersion state in an oil-based drilling fluid and, to some extent, decide the permeability of the mud cake formed on the wall. The overall performance of an oil-based drilling fluid depends partly on distributions of particle size and morphology. The micromorphology of the three weighting agents is scanned by using field emission scanning electron microscopy (Hitachi-SU8010) to observe the morphology and size of the particles, as shown in figures 5–7.

As can be seen from figure 5–7, the three weighting agents have different size and morphology features. For all the three agents, the distribution of single particle size is the same as the result shown in the particle size analysis. Superfine manganese ore particles have a more centralized distribution than submicron barite particles. Seen from the morphology, all the three weighting agents have apparently irregular structures. For standard barite, the range of the size is larger and the particle surface is coarse, on which there are some fractures. Some studies have already demonstrated that standard barite will become crushed under high pressure. For submicron barite, its morphology is quasi-circular and its size falls in the range of 0.8–4.0 µm. The quasi-circular morphology and the small size cause the particles to be unstably dispersing in the oil phase. At a low rotating speed, the particles are more likely to aggregate and the shear force of the drilling fluid will therefore increase, showing a higher apparent viscosity. For superfine manganese ore, the size range is also narrow, but there are small particles with a size less than 1.0 µm. Its morphological structure is longer, which is much different from the quasi-circular morphology of submicron barite. However, the long structure has some negative effect on the density of the mud cake.

### 4.2. Filtration loss and wall-building property of oil-based drilling fluid

**Figure 8.**
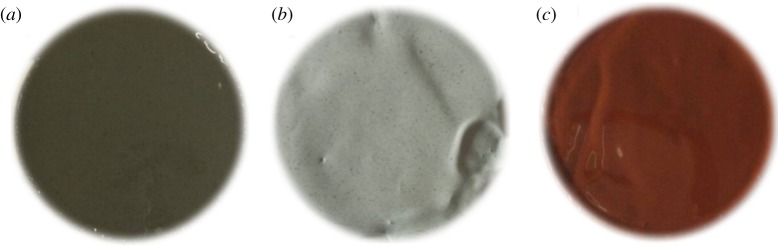
Appearance of mud cake. (*a*) Standard barite; (*b*) submicron barite; (*c*) superfine manganese ore.

Filtration loss and wall-building property is a core function of a drilling fluid and has great effect on the stability of the borehole wall of unstable stratum. For an oil-based drilling fluid, the filtration loss and wall-building property reflects mainly in the filtration loss rate and mud cake quality. If the filtration loss rate is too high, then the friction between fractures in the stratum is small and the stability of the borehole wall will therefore be affected. In addition, the large amount of the fluid filtrated into the stratum can cause formation damage such as emulsion plugging and liquid-phase entrapment. A poor control of filtration loss can also generate a thick mud cake, which may bring about a differential pressure sticking accident and, consequently, affect the accuracy of the downhole test. The solid-phase particle is an important tool to control the filtration loss of an oil-based drilling fluid, while the weighting agent is the major source of solid-phase particles. Therefore, the filtration loss and wall-building property of different amounts of weighting agents has been assessed with respect to high-temperature and high-pressure filtration loss and mud cake permeability, as illustrated in tables 5 and 6. Figure 8 shows the formed mud cakes.

**Table 5.** Filter loss (HPHT) of OBM with different weighting agents.

	filter loss with HPHT (ml)			
dosage (g)	standard barite	submicron barite	superfine manganese ore	compound (1 : 1)
200	1.6	2.2	2.0	1.2
400	2.0	2.8	2.6	1.4
600	2.4	3.2	3.4	1.5

**Table 6.** Mud cake permeability of OBM with different weighting agents.

	mud cake permeability (10^−3^ µm^2^)			
dosage (g)	standard barite	submicron barite	superfine manganese ore	compound (1 : 1)
200	0.9542	2.4369	2.2256	0.3698
400	1.4523	6.8585	5.3675	0.6847
600	2.8965	9.1385	10.6984	0.9873

The experimental results suggest that: with increasing the amount of the weighting agent, the filtration loss of the drilling fluid also increases and the permeability of the mud cake increases accordingly. Under the condition that the amount of the weighting agent is the same, the mud cake formed by submicron barite and superfine manganese ore is thick and porous, so the effect of reducing filtration loss is not good and the penetration rate is high. Factors that have an impact on the permeability of the mud cake include the size, particle size composition and concentration of solid-phase particles in the drilling fluid. For the drilling fluid containing submicron barite and superfine manganese ore, the quality of the mud cake is poor and the penetration rate is high. This is because although submicron barite and superfine manganese ore particles are small, the size range of submicron barite particles is narrow and the size composition is not good. As a result, pores of the mud cake cannot be well filled during the process of filtration loss. Consequently, the rate of filtration loss is high and the quality of the mud cake is poor. In comparison, the quality of the mud cake formed by standard barite particles is better. The size range of standard barite particles is wide enough for the formation of a multilevel bridge. Compared with submicron barite and superfine manganese ore, standard barite has better mud cake quality and the permeability of the mud cake is also lower. Generally speaking, standard barite has the best filtration loss and wall-building property and, therefore, is applicable for the drilling fluid used in a stratum with high water sensitivity or sandstone formation with high permeability. For submicron barite and superfine manganese ore, the filtration loss and wall-building property is poor, so they should be carefully adjusted and controlled in usage. The high-temperature and high-pressure filtration loss rate and the mud cake permeability of the compound are better than those of a single agent. This is because the multilevel bridge can form a more dense mud cake and the filtration loss and wall-building property is also better.

### 4.3. Acid dissolution efficiency of mud cake

Acidification is a common technology for increasing the production in mining in dense and unconventional oil and gas reservoirs and is also an important step in protecting the reservoir. It can be used to open the contaminated area around the wall of the borehole in the target reservoir, with a good effect of increasing the production. The weighting agent is a major source of solid-phase particles in an oil-based drilling fluid. The acid dissolution efficiency of the weighting agent is of great importance to help recover the permeability of the reservoir that has been contaminated by the drilling fluid. In this article, the acid dissolution efficiency of mud cakes formed by different weighting agents are assessed (table 7). We used 15% HCl to carry out the experiment, and HCl was of a pure level. The experimental procedure for acid dissolution of the mud cake: (i) dry the mud cake of different weighting agents at 50°C and take out the same gram number *m*_1_; (ii) put the mud cake into a porcelain bowl and add 15% HCl to soak for 10 h; (iii) filter the soaked pottery bowl with a 100 mesh screen and dry the residue at 50°C, weighing *m*_2_; (iv) compare the quality of mud cake before and after the experiment; the acid dissolution rate of the mud cake is (*m*_1_ − *m*_2_/*m*_1_) × 100%. The assessment suggests that: for the barite weighting agents, the acid dissolution efficiency is lower than 4%; for superfine manganese ore, the efficiency is above 97%; and for the compound of standard barite and superfine manganese ore that are mixed at a ratio of 1 : 1, the efficiency is as high as 50%, also showing a good effect of acid dissolution.

**Table 7.** Acid dissolution efficiency of OBM mud cake with different weighting agents.

	acid dissolution efficiency of OBM mud cake (%)			
dosage (g)	standard barite	submicron barite	superfine manganese ore	compound (1 : 1)
200	2.53	1.69	97.45	48.36
400	3.69	2.36	99.03	52.05
600	2.17	2.45	97.89	47.28

### 4.4. Mud cake lubricity

A weighting agent can be used to increase the density of an oil-based drilling fluid. However, high density can cause a series of problems such as high solid content, high viscosity and high friction coefficient. The lubrication performance of an oil-based drilling fluid can be assessed by using a coefficient. In our experiment, the mud cake viscosity coefficients of the three weighting agents and the compound agent are tested, and the results are listed in table 8. On the whole, the mud cake viscosity coefficient has increased with increase in the amount of weighting agent, as illustrated in table 8. For submicron barite and superfine manganese ore, however, the viscosity coefficient decreases when the amount increases from 200 to 400 g. Later, the viscosity coefficient increases quickly with continuously adding the two agents. For the compound agent, the viscosity coefficient has changed slightly. Causes for this trend include the following: (i) The particle size of standard barite is relatively large and the particles are not regular. Besides, the viscosity coefficient is large even at the beginning. As more large particles have been added into the fluid, the surface roughness of the mud cake keeps increasing and the viscosity coefficient becomes larger slowly. (ii) Submicron barite and superfine manganese ore are relatively small. At an amount of 200 g, most particles accumulate on the filtering surface, forming an uneven and thin mud cake, and the viscosity coefficient is small at the beginning. After an additional 200 g of weighting agent is added into the drilling fluid, a smooth mud cake is formed by those superfine particles and the viscosity coefficient becomes smaller. At an amount of 600 g, however, the two weighting agents are denser in the fluid, and the interactive force between particles promotes the aggregation of those superfine particles into larger ones. In consequence, the viscosity coefficient of the mud cake increases quickly. (iii) For the compound agent of standard barite and superfine manganese ore that are mixed at the ratio of 1 : 1, the superfine manganese ore particles and the large standard barite particles accumulate evenly on the filtering surface, forming an even and dense mud cake. In this process, the force between superfine manganese ore particles becomes weak, and the coarse surface caused by the large standard barite particles is filled with superfine manganese ore particles, so there is only a slight change of the viscosity coefficient. Seen from the perspective of the viscosity coefficient, standard barite is difficult to be applied in deep and ultra-deep wells because of its poor lubrication performance, which is a challenge for engineering application. For superfine particles, however, the problem of aggregation can be a limitation on application. The experiment results indicate that the compound is more favourable for improving the lubricity of the drilling fluid.

**Table 8.** Viscosity coefficient of OBM mud cake with different weighting agents.

	viscosity coefficient			
dosage (g)	standard barite	submicron barite	superfine manganese ore	compound (1 : 1)
200	0.1495	0.0699	0.0612	0.0524
400	0.1673	0.0437	0.0524	0.0524
600	0.1944	0.2217	0.1944	0.0612

### 4.5. Analysis of sedimentation stability

Sedimentation stability is a basic index to assess an oil-based drilling fluid system. The weighting agent can disperse and suspend well in an oil-based drilling fluid if the sedimentation is stable. In most cases, however, an oil-based drilling fluid has some problems relating to sedimentation stability, which is one of the two technological problems of an oil-based drilling fluid. In our experiment, the sedimentation of three different weighting agents in an oil-based drilling fluid has been observed to figure out the differences of the three agents, so as to provide some reference for practical application. The formula for the experiment is: oil base + 1.0% organic soil + 1.5% primary emulsion + 2.1% secondary emulsion + 20% CaCl_2_ brine + 2.5% fluid-loss agent + 2.5% lime + 0.5% wetting agent + 600 g weighting agent. The total amount of the compound agent that is mixed at the ratio of 1 : 1 is 600 g. In the experiment, the fluid to be tested is poured into a 500 ml measuring cylinder. The difference between densities of the upper and the lower oil-based drilling fluid is measured at room temperature after 12 h and 24 h, respectively. The experimental results are listed in table 9.

**Table 9.** Assessment of sedimentation stability with different weighting agents.

600 g weighting agent	density difference after 12 h (g cm^−3^)	density difference after 24 h (g cm^−3^)
standard barite	0.03	0.06
submicron barite	0.02	0.03
superfine manganese ore	0.03	0.07
standard barite + submicron barite	0.03	0.05
standard barite + superfine manganese ore	0.04	0.07
submicron barite + superfine manganese ore	0.03	0.04

The experiment of sedimentation stability shows that: the suspension of particles in the oil-based drilling fluid is not as good as that in the water-based drilling fluid. Although the change of the sedimentation stability is acceptable in engineering applications, the measured density is about 1.7 g cm^−3^ when 600 g of weighting agent is added into the fluid, which is lower than the density of 2.0 g cm^−3^, which is commonly required in engineering applications. In addition, the measurement is performed at normal temperature and pressure. In fact, basic properties of the oil-based drilling fluid will change greatly at high temperature and pressure. The above result indicates that the sedimentation stability should be carefully investigated. If fast sedimentation appears, it is not good for protecting the reservoir.

### 4.6. Rheology-control mechanism

An oil-based drilling fluid is often used in an environment with a complex structure such as a shale formation, deep well or ultra-deep well, where the wall of the borehole is not stable. Generally, the density should be higher than 1.5 g cm^−3^. In comparison with a water-based drilling fluid, the rheology-control mechanism for an oil-based drilling fluid is much different. For drilling fluid with low density, the rheology-control technology has been well developed, but for drilling fluid with high density, the adjustment and control of rheology is still a technological challenge, and the weighting agent is the core factor that has an effect on rheology control of an oil-based drilling fluid [27,28].

The solid content in the oil-based drilling fluid with high density is very large, and there is a high probability of the contact of solid-phase particles. So, the major force resistant to the flow of the drilling fluid is the friction between solid-phase particles. The viscosity of the drilling fluid system with high density comprises three parts: non-structural viscosity, structural viscosity and friction between solid-phase particles. As the number of solid-phase particles (coming mainly from the weighting agent) increases, the viscosity is more affected by the friction between the particles. With the interaction between the particles at a certain concentration, a force chain will be formed, which makes the rheology control more difficult. The weighting agent is the major source of solid-phase particles. Therefore, weighting agent is the main content for studying the rheology control of the oil-based drilling fluid.

Stribeck conducted a study on friction between particles and developed a curve named the ‘Stribeck curve’, which describes the friction coefficients of particles in different drilling fluids [29,30]. These can be broadly categorized as: hydrodynamic lubrication, mixed lubrication and boundary lubrication. During the stage of hydrodynamic lubrication, the spacing between particles is large, which falls in the range of 1–100 µm. This exists mainly in drilling fluid with low density. As the particle concentration increases, the friction between particles becomes larger and there is a coexistence of hydrodynamic lubrication and particle friction. In this stage, the spacing between particles is 0.1–1.0 µm. After the concentration of the weighting agent particles reaches a certain level, the relative motion of the particles will result in an increase of the friction coefficient and can cause a sharp drop of the effect of hydrodynamic lubrication, during which the spacing between particles falls in the range of 1–100 μm [31]. The changes in the friction caused by increasing the solid-phase particles in the three stages have apparent signs in the oil-based drilling fluid. In the stage of hydrodynamic lubrication, the oil–water interface of the system is stable, and the hydrophilic weighting agent particles can evenly disperse in the oil-based drilling fluid under the effect of the wetting agent, which contributes to the good rheology of the drilling fluid. When the number of the weighting agent particles increases, the stability of the oil–water interface is broken by the particles that have not been completely wetted, and the interactive force between the particles becomes stronger, which makes it difficult to control the rheology. After the concentration of the weighting agent particles reaches a certain level, the oil–water interface is destroyed, and particle sedimentation and hydrophilic aggregation then appear in the oil-based drilling fluid. As a result, the friction between particles becomes stronger and the rheology becomes unstable.

According to both the assessment of the rheology of different weighting agents and the analysis of the particle size and morphology of the weighting agents, the apparent viscosity of the oil-based drilling fluid that is added with the standard API barite falls in a normal range. However, when the fluid is added with a micro-nano weighting agent, i.e. submicron barite and superfine manganese ore, the viscosity increases sharply. If the viscosity increases in a normal range, the drilling fluid can take away more rock debris, but if the viscosity increases too fast, the drilling efficiency will decrease. The rheology can be controlled if the friction between particles becomes smaller, which can be realized by increasing the spacing between the particles through changing the size of the weighting agent. Therefore, the compound made of different sizes of particles is an effective way to control rheology.

The surface of the weighting agent is generally hydrophilic. A surface-active agent or surface modification can be purposely used to make sure that the particles of the weighting agent are evenly dispersing in the oil-based drilling fluid, which is a key to controlling the rheology. In addition, different material properties can be properly used to enhance the control of the rheology of the oil-based drilling fluid, for example, the changes of the friction caused by different particle shapes.

### 4.7. Damage-control mechanism

For either an oil-based or water-based drilling fluid, the damage to the reservoir comes mainly from both physical and chemical effects of the particles and the fluid that intrude into the reservoir. Therefore, reducing the intrusion of the oil-based drilling fluid is the key for damage control, while the most direct and the most efficient way to reduce the intrusion is to control the quality of the mud cake. There are two patterns to reduce the intrusion of the fluid and particles: extremely short formation time of the mud cake and extremely low permeability of the mud cake [6].

The formation of the mud cake is the result of the sedimentation of solid-phase and other particles captured in the permeation channel in the stratum, which depends on some physical and mechanical effects, such as inertia, diffusional sedimentation, gravitational sedimentation and electrostatic interaction. The mud cake is formed in two stages: the dynamic formation during the drilling process and the static formation after the drilling is stopped. The static filtration loss rate is smaller than the dynamic filtration loss rate, and the static mud cake is thicker than the dynamic mud cake. The fluid loss and wall-building property of the drilling fluid gradually becomes worse as the content of the weighting agent increases. This is because the solid-phase particles break the original particle composition in the fluid. As a result, the mud cake with extremely low permeability will not be formed. Besides, the rheology also becomes worse, which prolongs the formation of the mud cake.

In the oil-based drilling fluid without adding bridging particles or a reservoir protection agent, the solid-phase particles come mainly from organic soil, weighting agent and a small amount of fluid loss agent. Generally, the size of organic soil is about 0.2 µm, the size of standard barite is 10–50 µm, and the sizes of submicron barite and superfine manganese ore are 1–3 µm. There are two causes for the poor quality of the mud cake in an oil-based drilling fluid with high density: (i) The difference between the sizes of the standard barite and the organic soil particles. Consequently, the composition of particle sizes is not appropriate. (ii) In the mud cake, the number of large standard barite particles is much higher than the number of organic soil particles.

**Figure 9.**
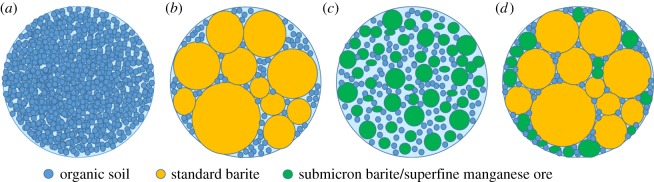
OBM mud cake formed by different solid-phase particles. (*a*) Organic soil; (*b*) standard barite + organic soil; (*c*) submicron barite/superfine manganese ore; (*d*) standard barite + submicron barite/superfine manganese ore.

To describe the quality of the mud cake, the weighing agent particles and the organic soil particles in the oil-based drilling fluid are simply seen as round particles, as shown in figure 9. Figure 9*a* is the mud cake formed by the matrix of the oil-based drilling fluid without adding any weighting agent. Particles with a size of 0.2 µm can accumulate at superfine pore throats to form a dense mud cake, which is efficient to control the filtration loss rate. But when the pore throat and fracture are larger, then the quality of the formed mud cake is poor. Figure 9*b* is the mud cake formed by the barite and organic soil, in which the particle size composition is 0.2 µm and 10–50 µm. The spacing between the two different sizes of the particles is large, and, in the drilling fluid with high density, the number of the organic soil particles is not big enough to form the bridge, which, therefore, cannot result in the formation of a dense mud cake. Figure 9*c* is a dense mud cake formed by 1–3 µm submicron barite and superfine manganese ore particles and 0.2 µm organic soil particles. Likewise, the number of organic soil particles is small, and the drilling fluid containing a superfine weighting agent is affected by many factors. Furthermore, the apparent viscosity is too large, which limits the use of superfine weighting agents in a large amount. Consequently, the quality of the mud cake formed by the particles of the two small sizes is affected. Figure 9*d* shows the mud cake formed by standard barite, submicron barite and superfine manganese ore, which have three levels of particle size, forming a multilevel particle size composition of ‘bridging by large particles—bridging and filling by medium particles—filling by small particles’. For each level, the particle size follows the normal distribution, which makes the particle size composition more reasonable. In addition, if the particles of the three sizes are mixed in a reasonable ratio, the properly increased viscosity is more favourable to take away solid-phase particles and improve the rheology of the oil-based drilling fluid. Of course, a reservoir protection agent, bridging particle and other treating agents can be used in accordance with practical conditions to refine the particle size composition, so as to further reduce the intrusion of the drilling fluid and particles and also protect the reservoir.

Seen from the perspective of reservoir protection, the reservoir shall be plugged quickly and tightly and the permeability of the reservoir shall be efficiently recovered after completion of the well. Measures to recover permeability of the reservoir include natural flow-back of solid-phase particles under the formation pressure, perforation and acid pickling, etc. [32]. Natural flow-back can be realized in some strata where the formation pressure is high. However, due to the complexity of an oil and gas reservoir, the efficiency of natural flow-back is not high and, therefore, some other flow-back measures are required. Perforation is a common measure to open an oil and gas reservoir that has been contaminated. But the perforation by an ordinary perforating gun is not deep enough, which cannot help to completely recover the permeability of the reservoir. Currently, acid pickling is the key measure to improve reservoir permeability. In most cases, acid solution is injected after perforation to pickle the acid-soluble mud cake, meanwhile extending a part of fractures under a high pressure, so as to further improve the permeability. As shown in the experiments, superfine manganese ore has a high acid dissolution efficiency and can form a part of an acid-soluble mud cake if compounded with standard barite particles and, under the washing effect of the acid solution, can recover the permeability by washing the dense mud cake in the fracture. Therefore, a proper amount of acid-soluble weighting agent can greatly protect the reservoir.

Generally speaking, the weighting agent for an oil-based drilling fluid shall be selected in accordance with practical geological conditions. Standard barite has a cost advantage. Submicron barite is suitable for reservoirs that have higher requirements on viscosity and can reduce the damage by using less treating agents. Superfine manganese ore is more protective for reservoirs, which is why it has been used in some blocks. The assessment of the weighting agents has focused largely on studying the effects of the agents on the oil-based drilling fluid, by which we have figured out the damage caused by the agents, i.e. the damage to the reservoir is influenced by material, particle size distribution and surface properties of the weighting agents. In applications, one or several compounds can be used in accordance with practical conditions to meet engineering requirements and also protect the reservoir.

## 5. Conclusion

(1) The rheological and suspension stability are two technical problems that cannot be solved well in an oil-based drilling fluid. The weighting agent is the core factor affecting the two properties. The rational selection of the weighting agent can improve the stability of the oil-based drilling fluid.
(2) The particle size and morphology of the weighting agent greatly affect the rheology of the oil-based drilling fluid. The particle size of most standard barite particles is above 10 µm, and submicron barite and superfine manganese ore are basically distributed in the range of 1–3 µm. The morphology of standard barite is irregular; submicron barite particles are quasi-circular structures; and superfine manganese ore particles are longer. The differences in both particle size and morphology have brought about changes in filtration loss and wall-building property, and the lubricity and sedimentation stability of the oil-based drilling fluid. A proper utilization of these differences can improve the rheological stability of the oil-based drilling fluid.
(3) The particle size, morphology, surface wettability and acid solubility of the weighting agent affect the reservoir protection performance of the oil-based drilling fluid. The degrees of the damage to permeability caused by the three weighting agents are higher than 50%, but when the acid-soluble superfine manganese ore particles are added into the oil-based drilling fluid after the acid dissolution, the damage degree can be controlled below 10%.
(4) For the oil-based drilling fluid, rheology control and damage control are complementary to each other. To improve the rheological stability of the oil-based drilling fluid and protect the reservoir, we should take full consideration of the properties of both the drilling fluid and the weighting agents, rationally design the particle granular composition, make use of acid dissolution, quickly form the mud cake with extremely low permeability and efficiently remove the mud cake.

## Data accessibility

This paper has no additional data.
